# Concussion Reporting and Safeguarding Policy Development in British American Football: An Essential Agenda

**DOI:** 10.3389/fspor.2021.671876

**Published:** 2021-06-04

**Authors:** Eleanor Travis, Claire Thornton, Andrea Scott-Bell

**Affiliations:** ^1^Department of HE Sport, Hartpury University, Gloucester, United Kingdom; ^2^Department of Sport, Exercise and Rehabilitation, Northumbria University, Newcastle upon Tyne, United Kingdom

**Keywords:** American Football, British Universities and Colleges Sport, player welfare, medical provision, British American Football

## Abstract

The objective of this study was to examine concussion reporting and safeguarding policy in British American Football (BAF). Data were collected *via* an online survey tool. The data presented are part of a broader study that examined injury profiles, concussion reporting behaviors, and medical provision in BAF. Concussion-like symptoms were found in over half (58.8%) of the participants. Of those, 36.4% reported they had previously been formally diagnosed with a concussion while playing BAF. Just under half of the participants (44.7%) had suspected they had had a concussion, although it was not formally diagnosed, and 23.5% of the participants had previously hidden concussion symptoms. Fifty-eight percent of the teams reported they did not have a regular game-day medic, with a range of hired medical personnel who attended the games. Prominent barriers to hiring a medic included budget, institutional support shortfall, and lack of medic reliability and game knowledge. BAF is a developing sport with a clear vision for growth of participation. Yet, the current concussion and medical provision policies do not address the sport's welfare needs. Through discussion of these policies in the context of this study's findings, we highlight vital areas of concern in policy and practice that the British American Football Association needs to address in their medical and concussion policies.

## Introduction

American football (AF) is one of the most popular sports in the United States (US) with ~5.16 million tackle players and 6.57 million non-contact flag football players in 2018 (Statista., 2021a). The sport regularly attracts numbers of around 500,000 spectators per team across the National Football League (NFL) season (Statista., 2021b). In the United Kingdom (UK), the game is less popular yet growing. According to the UK national governing body, the British American Football Association (BAFA), there were 462 registered teams in the 2019/2020 season.

American football was first popularized in the UK in the early 1980s (Maguire, 2011), and soon, a league was developed under the title of the American Football League (UK) (Needham, n.d.), which was replaced by BAFA in 2010 (Crawford, 2016). AF was first played at the university level in 1985 as a four-team league (Bayram et al., 2020). However, the game is now played under the British Universities and Colleges Sport (BUCS) umbrella. In the 2019/2020 season, this league encompassed over 80 teams (BUCS, 2021).

Following the recent changes to the governance of BAFA, a “10-year vision to professionalize British American football and inspire people to play the game” was outlined (BAFA, 2020). This vision statement includes (but is not limited to) “accessible, inclusive, safe and enjoyable” participation growth across all levels, long-term athlete development, and supporting the quality and quantity of game-day staff (BAFA, 2020). As part of this professionalization, BAFA has developed a number of policies, including medical provision, and concussion policies to support the safe participation of players.

The 2017 medical policy for tackle AF highlights five key areas for minimum standards:

(1) The professional body the first aider is registered to.
(2) The first aider should not be a team member.
(3) A risk assessment should be carried out by the first aider.
(4) A “suitable” first aid kit which is “approved by the professional practitioner” must be available.
(5) A telephone with signal should be available.

The policy states that these are minimum medical guidelines for teams to manage. Games will be suspended if these guidelines are not met. Additionally, BAFA's concussion policy states it is the shared responsibility of the player, coaches, and club management to oversee the recovery of an athlete following a concussion.

Unlike in the US where players are brought up playing the game from a young age (Findler, 2015), few UK athletes begin playing AF until later into their teens or early adulthood. Indeed, in their review of UK BUCS AF player injuries, Bayram et al. (2020) found that 39.5% of the players had no playing experience before the season began, and over 80% of the athletes started playing the sport at university that year. One potential risk of limited knowledge and experience of playing the game is increased injury incidence requiring medical intervention. Bayram et al. (2020) have reported that UK university AF injuries to be comparably different to US collegiate football injuries. Specifically, UK university players were found to have a greater risk of concussion and more severe injuries. Running backs and linebackers were found to have the highest injury rates, potentially due to their involvement in high-speed tackles (Edwards et al., 2018; Ward et al., 2018). Moreover, injury rates in offensive and defensive linemen were proportionally higher than US collegiate athletes (Badgeley et al., 2013; Dompier et al., 2015; Bayram et al., 2020). It is proposed that these findings are due to small roster sizes found in the UK game, meaning more game time for individuals. Furthermore, the provision of strength and conditioning, funding, coaching, officiating, and medical facilities is far from comparable to that of the US sport (Bayram et al., 2020).

AF exposes athletes to frequent collisions and high-velocity movement, placing the athlete at considerable risk of both musculoskeletal (MSK) and head injuries (Edwards et al., 2018). For example, during the 2019 NFL preseason and regular season, there were 224 diagnosed concussions (Battista, 2020), and during the 2012 and 2013 seasons, 262 concussions were reported in collegiate players (Dompier et al., 2015). Comparably, Bayram et al. (2020) reported three times the risk of concussion in UK university football to collegiate football in the USA. However, due to a significant shortfall of research in the British game, we do not know the extent of injuries within the British American Football (BAF) across all levels. More importantly, our knowledge of reporting behaviors, injury profiles, physiological demands, injury management protocols, and knowledge of these protocols among BAF players and staff continues to be sparse. As such, the concussion incidence rate could be higher. The tendency for players to underreport injury (Kroshus et al., 2015; Cranmer and LaBelle, 2018) is particularly concerning in light of the emergence of chronic traumatic encephalopathy (CTE) as a potential long-term effect of concussion (Omalu et al., 2010) and second-impact syndrome, the consequence of a second head impact which can lead to severe neurological consequences and even death (Jordan, 2013).

Concerns for the secondary effects of concussion have led to increased scrutiny from key game stakeholders regarding the safety of players. Indeed, media, sports fans, athletes, and academics have called for the abolishment of the sport, arguing that football is now too dangerous a game to play (Findler, 2015). In recent years, a decline in US youth football participation has been seen. This decline is reportedly due to concerns over head injuries sustained during participation (The National Federation of State High School Associations, 2019; Pielke, 2020). In light of this, the NFL and USA Football (USA's AF national governing body) have taken various steps to make the sport safer. These steps include implementation of baseline concussion tests and concussion evaluations that help medics determine whether or not a concussion has occurred (McDaniel, 2019). Additionally, there are now strict “return to play” policies at all levels of the sport for those athletes who have had a suspected concussion, and helmet-to-helmet hits have been banned. The “Heads Up Football” tackling program was introduced by USA Football and there is a limit on the number of full-contact practices that can take place in one season (Findler, 2015). However, at present, the British game has not taken the same steps to reduce injury and suppress the anxieties of those involved in its own game. Rather, it has signposted the aforementioned resources from USA Football which may not be suitable to the British game due to potentially different rates of injury compared to US AF (Bayram et al., 2020) and game-day staff provision. Furthermore, BAFA's minimum medical requirements provide basic life support only, rather than consider the inclusion of MSK injury and concussion care. This is evidenced in the concussion policy which is an educational document for the athlete (how to recognize concussion and safely return to play) rather than an informative medical policy for the medical practitioner. Yet, as seen in the aforementioned studies conducted in the UK, BAF players are at greater risk of MSK and head injuries (Bayram et al., 2020) than cardiac arrest. Thus, if the BAF game wishes to develop, steps should be taken to support player welfare in light of the data from its own population. This is particularly important when smaller team rosters mean greater game time which could lead to an increase in injury incidence and severity (Bayram et al., 2020).

## Methodology

### Study Design and Procedures

Data reported here are part of a broader study that examined the injury profiles, reporting concussion behaviors, and management of these injuries in BAF. The present data aimed to examine concussion reporting and safeguarding policy in BAF.

### Procedure

Ethical approval was granted by Hartpury University Ethics Committee prior to commencing the study. Potential participants were approached *via* email and social media (Twitter, Facebook, and Instagram) in order to reach participants nationally. Both non-validated surveys were made available *via* online surveys and the link was shared *via* previously stated methods. Consent to participate was required prior to proceeding with the questionnaires, and participants were guaranteed that neither their identity nor their team's identity would be disclosed.

The first questionnaire explored the profile of players across leagues in BAF, their injury status, and concussion reporting behavior. This questionnaire included sections on player demographics (14 questions), concussion history (6 questions), and reporting behavior (4 questions). Data were collected from 226 participants (mean age 24.0 ± 5.7) across the BAF leagues. Participants were excluded if they were under 18 years old. The majority (83.2%) of participants reported that they had played between 0 and 6 years of AF.

Table 1 shows the participants' profile across the leagues.

**Table 1:** Player profile.

Version of the game	Contact/tackle football	197 (87.2)
	Flag/non-tackle football	18 (8)
League	University football	102 (45)
	Senior men's league	74 (32.7)
	Women's league	26 (11.5)
	National program	16 (7.1)
	Youth league	8 (3.5)

*Brackets denote percentage of responses in each category*.

The second survey evaluated the medical provision and policy compliance in the 2019/2020 BUCS AF season and was comprised of 24 questions. This survey was completed by the BUCS club representative deemed most appropriate to complete the survey, e.g., general manager, head coach, or medic. Those who answered the survey participated in the sport from across the BUCS league.

Thirty-one teams completed this survey: premiership (19.4%, *n* = 6), division 1 (35.5%, *n* = 11), and division 2 (45.2%, *n* = 14).

In addition to the closed questions, optional commentary sections were included at the end of each section to allow participants to expand upon their experiences. A thematic analysis was conducted to highlight the most recurrent and prominent themes. Descriptive statistics were calculated for both questionnaires.

## Results

### Concussion

Concussion-like symptoms were reported in over half (58.8%, *n* = 133) of the participants. Of those 133, 36.4% (*n* = 82) reported that they had previously been formally diagnosed with a concussion while playing BAF, and 54.9% (*n* = 54) indicated that they had experienced more than one concussion. Of those who reported experiencing concussion-like symptoms, 37.8% (*n* = 17) reported they had experienced two concussions per playing career, and 15.6% (*n* = 7) reported they had experienced five or more concussions.

Just under half of the participants (44.7%, *n* = 101) reported they had suspected they had had a concussion; however, they were not formally diagnosed with one. Of these 101 participants, 52.5% (*n* = 53) reported this had been the case once, 28.7% (*n* = 29) reported their suspected concussion had gone undiagnosed twice, and 5.9% (*n* = 6) reported that their suspected concussion had gone undiagnosed five or more times.

Only 32.3% of teams reported conducting baseline concussion testing each new season and fewer (22.6%) carried this out with each new player who joined the team. One team commented that they have “no concussion training at all.”

### Concussion and Injury Reporting Behavior

When asked about their reporting behavior, the hiding of concussion/s from the coach or medical staff was reported by 23.5% (*n* = 53) of the participants. Yet more participants (62.8%, *n* = 142) reported to have previously hidden injury symptoms from coaches or medical staff. Out of those players who hid their injury symptoms, 59.9% (*n* = 85) downplayed the injury, 35.2% (*n* = 50) ignored the injury, and 4.9% (*n* = 7) denied any injury.

### Medical Personnel

Fifty-eight percent of BUCS teams reported they did not have a regular team medic. Of those who did have a regular team medic, 100% attended home games, 61.5% attended away games, and 7.7% attended training.

There were a range of medical personnel who attended BUCS BAF games. These included physiotherapists, graduated sports therapists, paramedics, St Johns Ambulance first aiders, and a sports rehabilitator. In the majority of cases (61.3%, *n* = 19), the highest qualification of the team medic was unknown by the club representative completing the survey.

Of the 61.5% of teams who did not have a regular medic who attended training, their medical provision was provided by a coach with first aid training (54.8%), a player with first aid training (32.3%), and “other” (12.9%, reported as facility/ground staff, students studying medical degrees, or coaches with first aid training), and 9.7% reported using an external paramedics company.

When teams were asked about their confidence in their medics' ability to safely remove a player's helmet and pads in an emergency (e.g., access to airways) thereby preventing inadvertent movement of the head or neck which could further compromise the athlete, 22.6% reported “somewhat confident” and 22.6% reported “unsure.” When asked about the confidence in their coaching staff to remove a player's equipment, 35.5% reported “confident” and 32.3% “somewhat confident.”

Positively, 83.9% reported that they would be interested in football-specific first aid training.

### Thematic Analysis

The commentary of participants on medical provision highlighted some common barriers to this including the expense of hiring, the shortfall of institutional support, and medical personnel with a lack of reliability and experience of the game. However, two university teams highlighted their successful use of an external business in providing medical provision, although it was acknowledged that this was uncommon. These themes will be explored further in the discussion.

## Discussion

This preliminary study is the first to look at concussion reporting and medical provision within the BAF league, providing a grounding for further research in the field. These findings highlight some key concerns within the sport which need to be addressed by key stakeholders. As such, the results will be discussed alongside BAFA's concussion and medical provision policies to explore the significance of these findings.

Under the current BAFA concussion policy and medical provision requirements, individual teams are responsible for implementing their own medical management plans, which only need to meet BAFA's minimum requirements, i.e., ensuring clubs have “adequate first aid cover.” This may result in compromises in player safety when management of medical provision is left to individual teams. Indeed, one participant commented that “all costs are met by competitors. They choose to afford minimal coverage” and another commented “I am happy with our coverage but for away games tend to have the bare minimum required.” There appear to be two issues highlighted here. Firstly, it is unfair and unsafe to place responsibility for deciding the level of medical cover on the athlete given that they may not understand the serious implications of injury and concussion (Guskiewicz et al., 2007). Secondly, the reliance on teams to self-manage their own medical provision creates disparity within the leagues and, from the comments made, could indicate that when teams are given minimal guidance in providing medical cover, teams choose the most affordable option: minimum medical requirements. However, since “[home] game management is responsible for the provision and suitability of medical facilities,” traveling teams have little/no power to influence the level to which the guidelines are met. Additionally, this finding leads us to question the degree to which teams conform to the current BAFA concussion policy and their interpretation of “adequate first aid.” Thus, without stronger central governance, there may be teams who will fail to protect the safety of the players. As Malcolm (2019) states, it is the sports governing bodies' ethical responsibility at all sporting levels to ensure coaches comply with concussion policy.

It is not uncommon for coaches to be involved in concussion management due to the rare presence of healthcare practitioners during practice (Follmer et al., 2020), yet they cannot be relied upon to manage concussion injuries due to their own responsibility for the team's success (Dillon, 2011; Partridge, 2014). Research also suggests that coaches expect players to willingly put themselves at risk of injury and continue to play injured for the good of the team (Malcolm and Sheard, 2002). Thus, putting the onus on teams to manage concussion is a risk to player welfare.

The diagnosis of sports-related concussion is perhaps one of the most challenging tasks facing sports medicine clinicians due to the uncertainty of biological markers and the need to rely heavily on the reporting of player symptoms (McCrory et al., 2017). The findings of this study indicate that 58.8% of the participants believed they had previously had concussive symptoms; however, 44.7% had not received a medical diagnosis. It is unclear whether these reported concussions occurred in training or during a game. As we see in the BUCS BAF game, there are inconsistencies in the medical provision at games and training. Further research should look to understand whether players who suspected they had a concussion chose not to disclose this or whether the inconsistencies in medical cover meant there was no one to disclose this injury to. This could also include investigations of the assessment of player's and game-day staff's knowledge of concussion signs and symptoms. Furthermore, baseline concussion assessment could be implemented to support diagnosis of suspected concussions (McCrory et al., 2017). The findings suggest that only 32.3% of teams carried out baseline concussion testing each season. At present, BAFA's concussion policy recommends the use of the Sports Concussion Assessment Tool version 3 (SCAT3) in the assessment of concussion only. However, as we understand that there are inconsistencies in medical provision, implementation of SCAT in a baseline capacity might support both players and medics in the assessment of concussion and graduated return to play (GRTP) process.

Current BAFA concussion policy states that all those involved in the game should be aware of the signs and symptoms of concussion to allow for early recognition. Yet, as BAF is an amateur sport, it cannot be presumed that all those involved in the game are familiar with indicators of concussion. Indeed, one participant noted they have “no concussion training at all.” Similar studies examining combat sport suggest that coaches are unfamiliar with recognizing concussion prior to implementation of educational programs and, instead, source information from unreliable sources (Follmer et al., 2020). With 54.8% of teams reporting that the coach is the designated first aider at training, it would imply that (in this setting) they are the primary source of concussion and injury information. Behavior such as this might exacerbate reliance on teams to manage concussion rather than on medical professionals.

All game-day staff (e.g., coaches, referees) have a duty of care to their players; therefore, it is their responsibility to report the suspicion of injury (including concussion) to the game-day medic. However, difficulty arises when the hired medical provision cannot be relied upon. The findings from this study suggest that BAF staff lack confidence in game-day medics, calling them “unreliable.” For example, one participant noted “Our AU [Athletic Union] try to use the same pool of people who gradually have developed a limited experience of the sport but subject to availability, it may sometimes be someone who has no experience of the sport or the sport-specific injuries.” This is most concerning for the player's welfare because a single diagnosed concussion can have considerable health implications such as a variety of neurologic and cognitive symptoms (Edwards and Bodle, 2014). Moreover, undiagnosed concussions are associated with higher postconcussion symptom scores and higher loss of consciousness rates with further incidences of concussion (Meehan et al., 2013). While BAF's status as a new and emerging sport may mean that there is only a small pool of practitioners with specific and appropriate training in this field who are able to support the game, withholding medical cover raises significant questions for player welfare and duty of care.

The British American Football Coaches Association (BAFCA) does not at present require coaches to have first aid certification, and current BAFA concussion policy simply encourages club personnel to complete first aid courses “appropriate to their role” (a statement which is open to interpretation). Yet, to rely on a first aid provision by a coach (with presumably no medical background) to recognize a concussion is unsafe. Moreover, there may be a conflict of interest given the coach's focus on performance. For example, one participant commented, “some players who were injured or had suffered illnesses felt pressured to play while recovering from injuries or while still being ill.” As evident in the findings, players are willing to hide concussion from game-day staff. Moreover, coaches may be reluctant to remove a player despite recognized signs and symptoms, due to the possible detriment to team performance (Dillon, 2011). Similar concerns are held with the 32.2% of teams who reported first aid provision coming through a player at practice sessions. Current BAFA policy advises that “clubs must ensure that they have adequate first aid cover available for all practice sessions where contact will take place,” yet what determines “adequate” is unclear. Furthermore, the policy does not stipulate whether this refers to both flag-football and tackle football, as concussion occurs in both versions of the game (Kaplan et al., 2013; Prien et al., 2018). Thus, current “policy” may be deemed a haphazard approach to medical cover than outline true policy directives.

Despite the current BAFA concussion policy which states that “players must remember their duty to inform their coach of their condition and any recurrence of it,” this study shows that a “headstrong” mentality (the willingness to conceal and play through injury; Liston et al., 2018) is present in BAF. The results of this early study suggest that players may have greater respect for concussion injury when compared with the reporting of musculoskeletal injuries, as players reported they are more likely to hide injury symptoms (62.8%), whereas only 23.5% reported hiding concussion. A possible explanation for this is the increased media coverage of both the long- and short-term implications of repeated head trauma (Gardner et al., 2014). Another reason could be likened to a copycat behavior from that seen in the NFL and other contact sports played in England such as rugby or the cultural practice of conforming to masculine norms (Kroshus et al., 2017) when playing through pain and injury, which is highly valued and positively reinforced (Atkinson, 2010; Fenton and Pitter, 2010).

Despite the threat that concussion poses to the national governing body (for example potential lawsuits from players seeking compensation for medical negligence), BAFA is yet to outline a detailed policy against this potential medicolegal action. Currently, BAFA policy on concussion has been taken from existing policies in other sports. The formation of a policy unique to BAF is required and should include consideration of the leagues, as well as a clear (GRTP) process for concussion. Yet, there are difficulties with this, for despite the sports understanding and vision to professionalize the game, there is a worry that if the policy is too detailed, participation in the sport will decrease. We understand from this study's findings that cost is a determining factor when considering the hiring of medical provision, indicating that some teams may already be financially stretched, particularly if membership numbers are low. However, we should ask the question as to whether we can compromise player safety for expense. Indeed, when research suggests that UK BUCS BAF players hold three times the risk of acquiring a concussion during a season compared with US American football athletes (Bayram et al., 2020), action should be taken to reduce this risk. If BAFA is capable and willing to enforce rules regarding playing (including during the COVID pandemic), then we would hope that similar emphasis could be taken to address concussion policy. This is particularly important at a time when collision sports are coming under criticism for their “contact” nature, but increasing participation and long-term athlete development is part of BAFA's 10-year development/strategic plan. Putting medical regulations in place that “do enough” but do not restrict play is required. For example, if more rigorous medical cover regulations were put in place that included sanctions for breach of minimum medical standards, this could help protect player safety. What this policy eventually covers requires an in-depth discussion, including how compliance will be monitored.

At present, the BAFA concussion policy has limited guidance for the medical practitioner with regard to GRTP following removal from the field due to concussion (i.e., who they should refer the care of the athlete to). Limitations in this current policy can be seen where teams have non-consistent medical personnel at training and games. The onus of the return to play (RTP) process is then placed upon the athlete themselves and/or the coach, neither of which may have the knowledge to safely go through the GRTP steps. As such, on game day, medical practitioners (unfamiliar with the team/athlete) may experience pressures from players who dispute their diagnosis or readiness to RTP (Malcolm, 2019). Furthermore, concussion symptoms are unique to the individual (McCrory et al., 2017). Thus, if teams have inconsistent medical support, the recognition of these changes in a player can be tougher if the practitioner is unfamiliar with their normal behavior, e.g., recognizing aggression in an athlete who is otherwise normally calm tempered. This places the medical practitioner in a difficult position and leaves them open to dispute with the athlete and coach about their removal from the game (Channon et al., 2020). It is here where we see that it is the initial diagnosis rather than the rehabilitation process that becomes an issue (McNamee and Partridge, 2013). Therefore, this may lead to practitioners feeling pressure about the decision to diagnose concussion in the first place, risking unethical practice (Partridge, 2014; Malcolm, 2019). As such, when forming future policy, serious consideration should be given to protect both players and practitioners.

Key findings suggest that only 32.3% of teams carried out baseline concussion testing each season, a method advised for interpreting suspecting postconcussion scores (McCrory et al., 2017) and advocated by other sporting bodies such as the National Collegiate Athletic Association (NCAA) (NCAA, 2021). Despite the literature which suggests that there are limitations to the Sports Concussion Assessment Tool's (SCAT) validity and utility, it may be of use in the GRTP of a player post-concussion (Yengo-Kahn et al., 2016). However, BAFA's concussion policy recommends the use of the SCAT-3 in the assessment of concussion only, suggesting that policy should be updated to include this new recommendation which would support player welfare.

BAFA states that one of its core missions is to develop a “safe and enjoyable player-focused environment” (BAFA, 2020). However, the current concussion policy comes with the caveat that it is the team's responsibility to manage concussion and its education. A regulatory body, established (in part) for the purpose of protecting the safety of its members and whose claimed mission is to promote safe play, appears to provide meager guidelines for its league to follow.

## Limitations

While this material provides an illustration of the incidence of concussion and current BUCS medical provision in BAF, this study is limited by comparisons across the different leagues and number of participating teams. However, these findings should not be discredited.

## Conclusions

To the authors' knowledge, this is the first study to provide an evaluation of BAF concussion reporting and safeguarding policy. The findings provide an illustration of the incidence of concussion and current medical provision in BAF and raise questions regarding policy suitability. Through discussion of this, we provide a grounding for further research in this field in support of policy development.

## Data Availability Statement

The raw data supporting the conclusions of this article will be made available by the authors, without undue reservation.

## Ethics Statement

The studies involving human participants were reviewed and approved by Hartpury University. The patients/participants provided their written informed consent to participate in this study.

## Author Contributions

All authors listed have made a substantial, direct and intellectual contribution to the work, and approved it for publication.

## Conflict of Interest

The authors declare that the research was conducted in the absence of any commercial or financial relationships that could be construed as a potential conflict of interest.
